# Assessing Knowledge of, and Attitudes to, HIV/AIDS among University Students in the United Arab Emirates

**DOI:** 10.1371/journal.pone.0149920

**Published:** 2016-02-25

**Authors:** Dalia Haroun, Ola El Saleh, Lesley Wood, Rola Mechli, Nada Al Marzouqi, Samir Anouti

**Affiliations:** 1 Programs, UNICEF Gulf Area Office, Dubai, United Arab Emirates; 2 Natural Science and public Health, Zayed University, Dubai, United Arab Emirates; 3 Preventive Medicine, Ministry of Health, Dubai, United Arab Emirates; Rutgers University, UNITED STATES

## Abstract

**Background:**

The Middle East and North Africa (MENA) region is among the top two regions in the world with the fastest growing HIV epidemic. In this context, risks and vulnerability are high as the epidemic is on the rise with evidence indicating significantly increasing HIV prevalence, new HIV infections and AIDS-related deaths.

**Objective:**

The aim of the survey was to assess HIV/AIDS knowledge and attitudes related to HIV/AIDS among a wide group of university students in the United Arab Emirates (UAE).

**Methods:**

In a cross-sectional survey, a total sample of 2,294 students (406 male; 1,888 female) from four universities in three different Emirates in the UAE were approached to take part in the study. Students self-completed a questionnaire that was designed to measure their knowledge and attitudes to HIV/AIDS.

**Results:**

The overall average knowledge score of HIV.AIDS was 61%. Non-Emirati and postgraduates demonstrated higher levels of knowledge compared to Emirati and undergraduate students respectively. No significant differences between males and females; and marital status were found. Eighty-five percent of students expressed negative attitudes towards people living with HIV, with Emirati and single students significantly holding more negative attitudes compared to non-Emiratis and those that are married respectively.

**Conclusions:**

The findings provide strong evidence that there is a need to advocate for appropriate National HIV/AIDS awareness raising campaigns in universities to reduce the gaps in knowledge and decrease stigmatizing attitudes towards people living with HIV/AIDS.

## Introduction

Although a marked diversity in HIV epidemiological patterns, trends and typologies can be found in it, the MENA region is among the top two regions in the world with the fastest growing HIV epidemic[1]. In this context, risks and vulnerability are high as the epidemic is on the rise with evidence indicating significantly increasing HIV prevalence, new HIV infections and AIDS-related deaths. According to the ‘UNAIDS Report (2014)’[1], more than 25,000 people got newly infected with HIV in 2013 marking a 7% increase between 2005 and 2013 and bringing the number of people living with HIV to 230,000 [CI: 160,000–330,000]. Simultaneously, there was an alarming increase of 66% in AIDS-related deaths in this region and up to 21,000 person died of AIDS-related causes in 2013[1].

Against this backdrop, the impact is particularly affecting children and adolescents. In this respect, in 2013, there were 2,300 [CI: 1,500–3,400] new infections among children [1]. Moreover, only 19% [12%–31%] of infants born to women living with HIV are tested in time; the pediatric treatment coverage rate is unacceptable with only 15% [CI: 11%–21%] of children living with HIV receiving anti-retroviral therapy (ART); and up to 1,600 children died of AIDS. During the same year, up to 5,400 adolescents (aged 10–19) got newly infected with HIV bringing the estimated number of adolescents living with HIV up to 20,000 in MENA countries [2]. This is further supported by a trend of increasing HIV incidence in the younger age groups, as well as, persistent (a) low awareness, knowledge and skills (e.g. % of Young Women aged 15–24 are sufficiently knowledgeable about HIV and AIDS is only 2% in Yemen, 3% in Iraq and Egypt, 5% in Palestine, 6% in Jordan and Syria, and 8.5% in Sudan); (b) increase in risky behavior, particularly among youth; and, (c) limited availability, access, and utilization of adequate youth friendly services and commodities (e.g. % of young women aged 15–24 who have been tested for HIV in the last 12 months and who know their results is 0.2% in Qatar, 0.5% in Tunisia, and 1.6% in Algeria) [3].

There is a limited number of studies assessing the knowledge and attitudes of university students on HIV/AIDS in MENA countries with available data from young people reported from Kuwait [4], Yemen [5], Turkey [6–7], Saudi Arabia [8], Iran [9] and Oman [10]. The population groups in these studies mainly included students from the medical or dental field for their ethical responsibility to provide appropriate and adequate treatment for people living with HIV. Findings of these studies, including among those in the medical field, revealed gaps in knowledge regarding HIV transmission together with intolerant attitudes towards people living with HIV.

It is particularly important that any regional group particularly vulnerable to HIV should have adequate knowledge about the disease so that they can help to protect themselves against possible risks. Despite the fact that young adults in the UAE are relatively well educated, with 80% continuing to higher education [11], many of them still have insufficient information on HIV in general. Many young people from the UAE travel widely and this, together with the rapid changes in cultural and religious values all contribute to a heightened risk of HIV. The first known study to estimate knowledge of HIV/AIDS among young people in the UAE was conducted in 2007 where data was collected from first year university students attending a National University in Al-Ain [12]. Similar to findings from other studies in the MENA region, they found misconceptions about modes of transmission and negative attitudes towards people living with HIV, with 75% of students considered to have low knowledge about HIV/AIDS and males were found to have better knowledge compared to females. A more recent study conducted in Ajman (UAE) in 2013 investigated dental student’s knowledge about modes of transmission of HIV/AIDS and their attitudes towards patients being treated [13]. Results also showed that students’ overall knowledge score was only 67% and that they had high levels of negative attitudes towards people with HIV.

Although previously-conducted studies in the UAE indicate that young people have insufficient knowledge on HIV/AIDS, these studies were limited to a small sample size, collected data from limited areas in the UAE, did not provide comparisons between population groups and therefore cannot be generalized to the UAE population. Hence, the aim of this study was to assess knowledge and attitudes related to HIV/AIDS among a large group of university students across different Emirates of the UAE to help inform the development of an adequate HIV/AIDS awareness raising component of a national HIV prevention program based on the current needs of the population. Differences in knowledge on HIV/AIDS and attitudes towards people living with the disease was also compared between sexes, nationality, marital status and education level.

## Material and Methods

### Study Design

The study was a descriptive cross-sectional survey designed to measure students’ knowledge and attitudes about HIV/AIDS among university students in the UAE. The actual data collection was carried out between March and September 2014.

### Sampling

The UAE federation consists of seven emirates (Abu Dhabi, Dubai, Sharjah, Ajman, Ras AlKhaimah, Fujairah, and Um Al Quwain). The UAE has only three governmental universities across the Emirates mainly attended by Emirati students in addition to several semi-governmental and private universities attended by both Emirati and non-Emirati students. All governmental universities and the main five private universities in the UAE were invited to take part in the study.

Using convenience sampling, the questionnaires were distributed to students using hard copies and collected by researchers. Only one university, due to having different campus locations, requested using both hard and soft copies (questionnaire sent electronically via email) to help increase student response rate. Students were randomly sampled from the university common areas including the library, canteen etc. Students were assured that all response were anonymous and that participation in the research was voluntary. No names nor any other identifying information were collected, and at no time could responses be linked to an individual.

### Data Collection Instrument

The questionnaire was designed with adequate consideration to UAE’s culture, approved by a committee formed of UNICEF and Ministry of Health experts, and piloted before the start of the study to ensure that all questions were clear and well understood. The questionnaire was made available in both English and Arabic languages.

Information was self-completed by students using a structured questionnaire that consisted of 13 closed-ended questions, presented in both English and Arabic languages, some of which were single response whilst others were designed to permit multiple responses. The questionnaire was divided into three main sections. The first section collected socioeconomic and demographic data such as year and subject of study, gender, marital status and nationality. The second section was used to assess students’ level of knowledge of HIV/AIDS and the third section evaluated measures of perceived attitudes of students’ towards people living with HIV/AIDS.

A measure of ‘knowledge score’ was then derived to indicate the overall level of knowledge university students had on HIV/AIDS based on the following 4 questions: 1. *Can a healthy looking person become infected with HIV*?, 2. *What are the* modes of *transmissions of HIV*, (with four correct options contributing to the score: (a) by having unprotected sex with HIV infected person, (b) during blood transfusion, (c) by sharing razors/ needles with HIV infected person, (d) from HIV positive mother to child during delivery or during breastfeeding), 3. *Can HIV can be cured*?, and 4. *What drug can be used to prevent serious illness in those infected with HIV*?

A measure of ‘stigma score’ was used to indicate the overall level of students’ beliefs that may lead to the development of stigmatizing behavior. The questions included (where responses deemed to provide evidence of a stigmatizing attitude): 1. *If you were to take HIV test*, *would you want anyone else to know the results*?*–‘No’); 2*. *What do you think the university should do with HIV infected personnel or students*?*–all four responses other than ‘They should be treated’ indicated stigmatizing attitudes which included isolated*, *reported to the authorities*, *sent away*, *and other); 3*. *Do you think that most people with HIV/AIDS have only themselves to blame*?*- ‘Yes’)*.

### Data Analysis

Data was analyzed using Stata version 12.0 (College Station, Texas). Data was checked for possible errors using standard methods (cross-tabs, maximum and minimum values for each variable). The analysis evaluated knowledge of HIV transmission, knowledge of more general aspects of HIV, and sources of knowledge about HIV. Descriptive statistics, including means, standard deviations, frequencies and percentages, were calculated for variables as appropriate. Student t-tests were used to examine any differences in continuous variables between males and females, students from the UAE and those of any other nationality, between marital status as well as between those that were undergraduate versus postgraduates. Chi-square statistics and logistic regression were used for categorical outcomes. Significance was set at p< 0.05.

### Ethics

Ethical approval was granted from UNICEF Gulf Area Office, and formal ethical approval was also obtained from the Ministry of Health, UAE (IRB: DFCM/09/02/14/022). Furthermore all four universities that participated in the study provided approval prior participating in the study. Participation in the study was on voluntary and unanimous basis.

## Results

### Participants Characteristics

A total of eight universities (3 governmental and 5 private) were invited to take part in the study, of which only one governmental (the largest university in the UAE) and three private universities agreed to take part in the study (50% response rate). The survey included participants from four universities geographically located across the three main Emirates in the UAE including Abu Dhabi (both the cities of Abu Dhabi and Al-Ain), Dubai and Sharjah, and included both UAE nationals and non-nationals from both genders. It also included students from different colleges both at the undergraduate and postgraduate levels.

Table 1 illustrates the study sample characteristics. A total of 2,294 students (406 male; 1,888 female) from four universities in the UAE responded to the survey, which was reflective of total student enrollment proportion of male versus female students. Of those, 1,359 (59%) were Emiratis, and 47 other nationalities were represented however the most common represented nationalities other than UAE included Syria, Jordan, and Palestine. Females were significantly more likely than males to be from the UAE (66% vs. 34%; p<0.001).

**Table 1:** Characteristics of Study Population.

	n	%
**Gender**		
** Male**	**406**	**17.7**
** Female**	**1888**	**82.3**
**Nationality**		
** Emirati**	**1359**	**59.2**
** Non-Emirati**	**924**	**40.3**
** Missing**	**11**	**0.5**
**Marital Status**		
** Single**	**2058**	**89.7**
** Married**	**189**	**8.2**
** Divorced**	**24**	**1.1**
** Missing**	**23**	**1**
**Year of Study**		
** First**	**711**	**31**
** Second**	**563**	**24.5**
** Third**	**449**	**19.6**
** Fourth**	**347**	**15.1**
** Postgraduate**	**214**	**9.3**
** Missing**	**10**	**0.4**
**University**		
** United Arab Emirates University**	**1590**	**69.3**
** Abu Dhabi University**	**268**	**11.7**
** University of Sharjah**	**220**	**9.6**
** American University in Dubai**	**216**	**9.4**
**Faculty**		
** Arts & Sciences**	**822**	**35.9**
** Business Administration & Information Technology**	**545**	**23.8**
** Engineering and Architecture**	**468**	**20.4**
** Medical and Health**	**182**	**7.9**
** Law**	**140**	**6.1**
** Food and Agriculture**	**127**	**5.5**
** Not stated**	**6**	**0.3**
** University College**	**4**	**0.2**

Almost all (90.2%) the respondents were undergraduates, 9.3% were postgraduate students and 0.4% did not specify their academic year. Most respondents (89.7%) were single, and 8.2% were married. Of the remainder, 1.1% were divorced, separated or widowed and 1% did not specify marital status. Arts and Sciences was being studied by just over one third (35.9%) of students, followed by Business Administration and Information Technology (by 23.8%) and Engineering and Architecture (20.4%).

### Participants Knowledge

The average total knowledge score for students was 4.3 out of 7 (61%). Only 4.3% students attained the maximum score (7; 100%). Table 2 provides a comparison of HIV/AIDS knowledge scores, by group. Females had slightly better knowledge than males on HIV but this was not statistically significant (61% vs. 59%; p = 0.06). Non-Emirati students scored 5.7% higher than Emiratis (64% vs. 59%; p<0.001), and similarly postgraduate students were significantly more knowledgeable than undergraduates by the same amount (66% vs. 60%; p<0.001). There was no significant difference in knowledge on HIV between single and married students (p = 0.16).

**Table 2:** HIV/AIDS Knowledge and Stigma Score by Group.

Type of score	Mean (% of correct answers)	SD	Mean (% of correct answers)	SD	Difference in means (95% CI)	p-value
**Knowledge**	**Males (n = 406)**		**Females (n = 1888)**			
	4.1 (58.6)	1.5	4.3 (61.4)	1.6	-0.16 (-0.33 to 0.004)	0.057
	**Emirati (n = 1359)**		**Non-Emirati (n = 924)**			
	4.1 (58.6)	1.5	4.5 (64.3)	1.6	-0.39 (-0.52 to -0.26)	<0.001
	**Single (n = 2058)**		**Married**^**‡**^ **(n = 213)**			
	4.3 (61.4)	1.6	4.1 (58.6)	1.6	0.16 (-0.06 to 0.38)	0.160
	**Undergraduate (n = 2070)**		**Postgraduate (214)**			
	4.2 (60%)	1.6	4.6 (65.7)	1.6	-0.37 (-0.59 to 0.15)	<0.001
**Stigma**	**Males (n = 406)**		**Females (n = 1888)**			
	1.5 (25.0%)	1.0	1.5 (25.0%)	0.9	-0.04 (-0.10 to 0.06)	0.414
	**Emirati (n = 1359)**		**Non-Emirati (n = 924)**			
	1.6 (26.7%)	0.9	1.3 (21.7%)	1.0	0.33 (0.50 to 0.41)	<0.001
	**Single (n = 2058)**		**Married (n = 213)**			
	1.5 (25.0%)	0.9	1.7 (28.3%)	0.9	-0.20 (-0.34 to -0.07)	0.003
	**Undergraduate (n = 2070)**		**Postgraduate (214)**			
	1.5 (25.0%)	0.9	1.4 (23.3%)	1.0	0.04 (-0.09 to 0.18)	0.521

Although many of the students knew the main modes of transmission of HIV, transfer of the virus from mother to child during delivery or breastfeeding was the least familiar with only 62% of students correctly identifying this to be true. Three-quarters of students were aware that HIV infection could be acquired through the use of sharing contaminated needle or razor blades while 85% and 86% respectively correctly thought that a contaminated blood transfusion or unprotected sex were potential sources of the virus. There was still considerable misconception, particularly about the belief that HIV could be acquired from a public toilet at 29% of students believing this to be a method of transmission (31.9% females and 18% males; p<0.001).

Table 3 shows students’ correct responses to the knowledge of transmission and treatment of HIV. In general, males and females had similar levels of knowledge about the methods of transmission of HIV/AIDS, with the exception that males were more likely than females (27% vs. 20%; p = 0.008) to believe that mosquito bites could be a mode of transmission, whilst females were more likely than males to incorrectly believe that HIV could be caught from using a public toilet (32% vs 18%; p<0.001). Compared with non-Emiratis, Emirati students had significantly lower levels of knowledge on the correct and incorrect methods of acquiring HIV. Almost twice as many Emirati compared with non-Emirati students (17% vs. 9.5%) incorrectly believed that HIV was not transmitted by having unprotected sex with an HIV positive person. There was no significant differences in responses between single and married students on the methods of transmission of HIV, other than married respondents being more likely than single students to incorrectly consider that touching an HIV positive person could lead to infection (13% vs. 9%; p = 0.032). Also, there was no significant difference in responses between undergraduate and postgraduate students, with the exception of postgraduate students being significantly more likely to identify HIV being transmitted by sharing needles with HIV infected persons (81% vs. 74%; p = 0.038) and from HIV positive mother to child during delivery or breastfeeding (61% versus 71%; p = 0.002)

**Table 3:** Students’ Correct Responses to the Knowledge of Transmission and Treatment of HIV by Group.

	Gender					Nationality					Marital Status					Level of Study				p
	Males		Females			Emirati		Non-Emirati			Single		Married/Divorced			Under-graduate		Post-graduate		
	n	%	n	%	p	n	%	n	%	p	n	%	n	%	p	n	%	n	%	
Identifying that a healthy looking person can have HIV	264	65.6	1270	67.9	0.35	886	65.7	641	69.9	0.037	1386	67.8	137	64.9	0.396	1377	67.1	149	70.3	0.397
Identifying HIV transmission by having unprotected sex with HIV infected person	347	85.5	1625	86.2	0.694	1128	83.1	836	90.5	<0.001	1778	86.4	175	82.2	0.096	1781	86.1	182	85.5	0.757
Identifying HIV transmission during blood transfusion	337	83	1615	83.6	0.191	1136	83.7	808	87.4	0.014	1758	85.5	176	82.6	0.266	1753	84.7	190	89.2	0.085
Identifying HIV transmission by sharing razors/ needles with HIV infected person	298	73.4	1417	75.1	0.488	979	72.1	729	78.9	<0.001	1550	75.4	152	71.4	0.213	1537	74.3	172	80.8	0.038
Identifying HIV transmission from HIV positive mother to child during delivery or during breastfeeding	243	59.9	1169	62	0.431	799	58.9	607	65.7	0.001	1267	61.6	133	62.4	0.825	1254	60.6	152	71.4	0.002
Identifying HIV cannot be transmitted by mosquito bites	298	73.4	1507	79.6	0.008	1082	79.7	707	76.5	0.07	1620	78.8	165	77.5	0.661	1629	78.7	161	75.6	0.294
Identifying HIV cannot be transmitted by touching HIV infected person	375	92.4	1707	90.5	0.256	1195	88.1	877	94.9	<0.001	1881	91.4	185	86.8	0.032	1881	90.9	191	89.7	0.534
Identifying HIV cannot be transmitted by sharing a meal with HIV infected person	359	88.4	1618	85.8	0.177	1147	84.6	821	88.8	0.004	1781	86.6	180	84.5	0.4	1786	86.4	181	85	0.601
Identifying HIV cannot be transmitted from public toilet	333	82	1285	68.1	<0.001	909	67	701	75.9	<0.001	1450	70.5	159	74.6	0.235	1452	70.2	157	73.7	0.305
Identifying HIV cannot be cured	143	38.1	768	44.8	0.018	510	41.6	397	46.4	0.035	827	44	78	40.6	0.401	808	43	100	50.8	0.041
Identifying ART is the correct treatment for HIV	51	23	272	29.2	0.067	167	25.5	152	31.1	0.039	290	28	32	29.9	0.735	275	26.5	43	39.8	0.005

In relation to the question on whether HIV can be cured, just over one quarter (26%) of students did not know whether HIV can be cured, 44% knew correctly that there is no cure and 26% did not know the answer. Comparing responses between groups suggests that males (38% males versus 45% females; p = 0.018), Emiratis (42% Emiratis versus 46% non-Emiratis; p = 0.035) and undergraduates (43% undergraduates versus 51% postgraduates; p = 0.041) were significantly less likely to correctly respond that HIV cannot be cured. No significant differences were found between those that were single versus those that were married. Overall, knowledge of HIV treatment was poor. When students were asked to correctly identify the drug used in HIV treatment, non-Emiratis (31% non-Emiratis versus 25% Emiratis; p = 0.039) and postgraduate students (40% postgraduate versus 26% undergraduate; p = 0.005) were significantly more likely to correctly identify that ART is used in treatment of HIV in comparison to Emiratis and undergraduate students respectively. No significant differences in responses to this question were found between sexes or marital status.

### Participants Attitudes

Overall, participants scored an average of 1.5 out of 6 (25%) on the stigma score. Emirati students had significantly higher stigma scores than non-Emiratis (27% vs. 22%; p<0.001) and married participants scored more highly than single students (28% vs. 25%; p = 0.0023). Only 15% of participants scored zero (i.e. no stigma) on the scale.

More than half (57%) of respondents said that they would not share the results of an HIV test with anyone. The proportion who would not share results varied across groups, with Emirati students reporting significantly greater reluctance than non-Emirati students (62% Emirati vs. 50% non-Emirati; p<0.001), and weak evidence that married respondents would be less willing than single students to tell someone else (63% married vs. 56% single; p = 0.067). No significant differences in responses were found between sexes and levels of study. Of those who said that they would tell someone about the results of an HIV test, 80% of students chose telling their parents. The majority of students indicated that they would tell more than one person including a sibling (37%), friend (18%), teacher (6%) or spouse (26%). No significant differences in stigma score were found between sexes and levels of study.

When students were asked what the university should do with HIV positive persons, over half (59%) of students responded that the university should treat infected personnel or students, while 28.7% responded that they should be isolated and 11% thought they should be sent away.

When students were asked whether HIV positive individuals had only themselves to blame, 36% responded yes, 38% did not think they were to blame and the remainder 26% were unsure.

Response patterns varied within some groups, with females being significantly more likely than males to think that HIV patients were to blame for their infection or to be unsure, whereas males were more likely than females to not think that there was an element of self-blame (p = 0.028). Emirati students compared to non-Emirati students were also far more likely to consider that HIV-positive persons had themselves to blame (40% vs. 29%; p<0.001). No significant differences between marital status and level of study were found in relation to this question.

## Discussion

This study found out that misconceptions about modes of transmission of HIV/AIDS, overall knowledge on HIV/AIDS and stigmatizing attitudes towards people living with HIV/AIDS continue to exist among university students in UAE, and vary by gender, nationality, marital status and level of study. Comparisons between universities or colleges were not carried out as universities in the UAE do not provide any education on HIV/AIDS to students (unless part of the Medical or Dental field). Results were similar to what was reported previously in other studies from MENA [3–10]. This study showed that 48% of students have low knowledge on HIV which was 27% lower than that reported in the UAE study that was carried out in 2007; which may indicate an increased level of awareness [12]. Comparisons however should be treated with caution as different assessment tools and population sample were used.

Students demonstrated some knowledge on the correct modes of transmission of HIV/AIDS however misconceptions are still present such as getting HIV from public toilets, mosquito bites, or touching an HIV infected person. This provides valuable information and guidance on how to create targeted messages and educational materials for young people in the UAE. Special attention should be given to Emirati students, especially males, who demonstrated the lowest level of overall knowledge scores on HIV/AIDS (mean score of 59%). Differences in scores between Emiratis and non-Emiratis could be attributed to differences in beliefs, cultures, religions and schooling, all which can have significant impact over the knowledge, attitudes and behaviors towards the disease.

Knowledge of the physical signs, cure and treatment of HIV/AIDS was low and should be addressed in order to improve the negative image of the disease. In a conservative society, HIV/AIDS is associated with taboos and the belief that HIV can only be transmitted though forbidden sexual relationships which further contributes to the stigmatization of people living with the disease. Although the overall stigma score was low, the stigmatizing attitudes of respondents, especially Emirati students, also represent a challenge as almost half of the respondents agreed with stigmatizing and discriminative actions. Anti-stigma communication strategies need special attention and should be directed to reduce negative attitudes towards people living with HIV. Stigma can be a serious barrier for the HIV/AIDS response and make young people less likely to get information on HIV, get tested or treated for HIV. The answers of the respondents reflected a strong moral stigma; 36% of the respondents agreed that HIV-infected persons had only themselves to blame and for fear of being stigmatized, over half (57%) would not share their test results with anyone and 29% of the respondents agreed that HIV-infected students should be isolated. Interestingly, young people reported disclosing their HIV status to family members rather than friends, which highlights the need to target students’ families with HIV/AIDS related messages. With only one quarter of our sample knowing that there is treatment for HIV, knowledge of treatment has been proven to greatly reduce stigma and discrimination against people living with HIV/AIDS as it diminished connotations of death and lethal disease [14].

Limited awareness on HIV/AIDS, lack of sufficient information on HIV/AIDS (even within schools and universities) and the reluctance of public health policy makers to carry out national HIV awareness programs due to the low national prevalence of the disease in the UAE, sensitivity the topic and cultural barriers all play a role in the findings of this study. In light of the increased prevalence of HIV/AIDS in the MENA region, preventive education is an integral and basic part of an effective and comprehensive combination HIV prevention program. Hence, effective knowledge and education programs should be mainstreamed across universities and schools in the UAE to prevent new HIV infections. More recently, UNICEF and Ministry of Health have joined forces and have trained group of ‘Peer Health Educators’ across a number of universities on raising awareness of HIV/AIDS within their communities; the effectiveness in this program is yet to be evaluated. Peer-led education program in promoting health has become increasingly popular in universities. Previous research has shown that peer-led HIV prevention programs improves the knowledge of students on HIV/AIDS and motivates them to have better attitudes towards people living with HIV [11,15,16]. Training peer educators might be more beneficial because of perceived trustworthiness of the information source and role modeling [17]. Results of this study will aid the development of appropriate National awareness raising messages to help reduce the gaps in knowledge and misconceptions on HIV/AIDS among young people.

## References

[1] UNAIDS, 2014 Fact Sheets, 2014.

[2] UNICEF, 2014 Statistical Update on Children, Adolescents and AIDS, 2014.

[3] UNICEF, Multiple Indicator Cluster Country Surveys (MICS), 2011–2014.

[4] Ellepola AN, Joseph BK, Sundaram DB, Sharma PN. Knowledge and attitudes towards HIV/AIDS amongst Kuwait University dental students. Eur J Dent Educ. 2011;15(3):165–171. 10.1111/j.1600-0579.2010.00652.x 21762321

[5] Badahdah A, Sayem N. HIV-related knowledge and AIDS stigma among college students in Yemen. East Mediterr Health J. 2010;16(8):901–906. 21469573

[6] Turhan O, Senol Y, Baykul T, Saba R, Yalçin AN. Knowledge, attitudes and behaviour of students from a medicine faculty, dentistry faculty, and medical technology Vocational Training School toward HIV/AIDS. Int J Occup Med Environ Health.2010;23(2):153–160. 10.2478/v10001-010-0008-5 20630832

[7] Bektas H, Kulakac O. Knowledge and attitudes of nursing students toward patient living with HIV/AIDS (PLHIV): a Turkish perspective. AIDS Care. 2007;19(7):888–894. 17712692 10.1080/09540120701203352

[8] Badahdah A. Stigmatization of persons with HIV/AIDS in Saudi Arabia. J Transcult Nurs. 2010;21(4):386–392. 10.1177/1043659609360873 20592063

[9] Tavoosi A, Zaferani A, Enzevaei A, Tajik P, Ahmadinezhad Z. Knowledge and attitude towards HIV/AIDS among Iranian students. BMC Public Health. 2004;4:17–26. 15157281 10.1186/1471-2458-4-17 PMC420470

[10] Al-Jabri A, Al-Abri J. Knowledge and attitudes of undergraduate medical and non-medical students in Sultan Qaboos University toward acquired immune deficiency syndrome. Saudi Med J. 2003;24:273–7. 12704503

[11] Barss P, Grivna M, Ganczak M, Bernsen R, Al-Maskari F, Agab HE, et al Effects of a Rapid Peer-Based HIV/AIDS Educational Intervention on Knowledge and Attitudes of High School Students in a High-Income Arab Country. J Acqui Immune Defic Syndrome. 2009;52(1) 86–98. 10.1097/QAI.0b013e31819c153f 19590431

[12] Ganczak M, Barss P, Alfaresi F, Almazrouei S, Muraddad A, Al-Maskari F. Break the silence: HIV/AIDS knowledge, attitudes, and educational needs among Arab university students in United Arab Emirates. J Adolesc Health. 2007;40(6):572–578. 10.1016/j.jadohealth.2007.01.011 17531765

[13] Premadasa G, Sadek M, Ellepola A, Sreedharan J, Muttappallymyalil J. Knowledge of and attitudes towards HIV/AIDS: a survey among dental students in Ajman, UAE. J Investig Clin Dent. 2013; 6(2):147–155. 10.1111/jicd.12080 24357612

[14] Bhatta D, Aryal U, Khanal K. Education: the key to curb HIV and AIDS epidemic. Kathmandu Univ Med J. 2013;11(42):158–161. 10.3126/kumj.v11i2.12493 24096225

[15] Thato R, Penrose J. A brief, peer-led HIV prevention program for college students in Bangkok Thailand. J Pediatr Adolesc Gynecol. 2013;26(1):58–65. 10.1016/j.jpag.2012.09.011 23332197

[16] Normala I, Lekhraj R, Zubaidah J, Azhar MZ. Effectiveness of peer-led education on knowledge, attitude and risk-behavior practices related to HIV among students at a Malaysian public university—A randomized controlled trial. Prev Med. 2012;55:505–510. 10.1016/j.ypmed.2012.09.003 22982947

[17] Kocken P, Voorham T, Brandsma J, Swart W. Effects of peer-led AIDS education aimed at Turkish and Moroccan male immigrants in The Netherlands. Eur J Public Health. 2001;11:153–159. 11420801 10.1093/eurpub/11.2.153

